# Hiccups and psychosis: two atypical presentations of COVID-19

**DOI:** 10.1186/s12245-021-00333-0

**Published:** 2021-01-20

**Authors:** Teresa Alvarez-Cisneros, Aldo Lara-Reyes, Stephanie Sansón-Tinoco

**Affiliations:** 1 Instituto Nacional de Geriatría Anillo Periferico, 2767 San Jerónimo, 10200 Mexico City, Mexico; 2 Medica Sur, Puente de Piedra 150 Toriello Guerra, 14050 Mexico City, Mexico

**Keywords:** COVID-19, SARS-CoV-2, Atypical manifestations, Psychosis

## Abstract

The WHO defines a possible case of COVID-19 as a person experiencing fever, cough, shortness of breath, and neurological signs including anosmia, ageusia, or dysgeusia. However, experiences from hospitals all over the world have shown that presentations vary widely. Some atypical presentations include cardiac, gastrointestinal, neurological, and cutaneous and while some are driven by the inflammatory response, others are a consequence of the hypercoagulable state. In our emergency department in a private hospital in Mexico City, we received two patients with very different symptoms on the same shift. Two previously healthy men in their 40s presented to the ER with very atypical manifestations of COVID-19. Neither of them complained of fever, cough, or shortness of breath. The first referred a 3-day history of hiccups that had not resolved with metoclopramide. The second presented with an acute episode of altered mental status. While the first case revealed lung involvement of the disease, the second case had a clean chest CT scan. These cases are relevant as manifestations of COVID-19 vary widely, especially in previously healthy young adults.

## Introduction and background

Fever, cough, shortness of breath, anosmia, ageusia, or dysgeusia are the most common symptoms of COVID-19. However, a wide variety of atypical presentations have also been described [1]. Some uncommon presentations are related to the hypercoagulable state, while others are a result of the inflammatory response. These manifestations can occur almost at any organ level. For example, cardiac manifestations include acute MI, myocarditis, arrhythmias, and pericarditis. Atypical heart manifestations are more common in individuals with previous heart conditions, but it seems that the high systemic inflammation and the angiotensin-converting enzyme 2 (ACE2) tropism are involved in this relationship and may cause manifestations in previously healthy individuals [2]. ACE 2 is also expressed in the gastrointestinal tract and up to 48% of patients present with gastrointestinal manifestations. They range from liver abnormalities such as mild to moderate liver injury with aminotransferase elevations and albumin decline, also diarrhea, nausea, and vomiting. Similar to the gastrointestinal tract, the nervous system also expresses ACE 2; thus, neuropsychiatric symptoms of COVID-19 occur in up to 36% of patients and are more frequent among those with severe disease [3]. The spectrum of neurological manifestations includes not only cerebrovascular disease and skeletal muscle injury, but also meningoencephalitis, encephalomyelitis, Guillain Barre, and perfusion abnormalities [4–6]. Psychiatric manifestations are wide and could be divided into those driven by the infection itself and those driven by stress and isolation related to the pandemic. Delirium has been suggested as the most common neuropsychiatric manifestation [7], but they also include psychosis, dementia-like symptoms, and affective disorders [8].

Considering these diverse presentations, it is relevant for the emergency department physicians to consider COVID-19 in unexplained manifestations of inflammatory disease or arterial and venous thrombosis at any organ level.

### Case 1

A 48-year-old man presented to the gastroenterologist with an episode of hiccups lasting at least 96 h. His past medical history only revealed an L5 surgical repair in 2005, and he denied allergies or recent travel. Aside from the hiccups, the patient recalled no other symptoms and denied abdominal pain, nausea, vomiting, diarrhea, chest pain, cough, dyspnea, or fever. He was started on ambulatory treatment with metoclopramide 10 mg every 8 h; however, the symptoms did not resolve.

He presented to the ER approximately 5 h later with the same complaint. His vitals showed a heart rate of 82 beats/min, respiratory rate 20 respirations/min, blood pressure 133/88 mmHg, oxygen saturation 93%, and temperature 36.7 °C. On the physical exam, the patient was alert and oriented with a normal affect and normal gait. The exam revealed no dermatological lesions and benign head, ear, nose, and throat exam. His chest showed a normal diameter. However, lung auscultation revealed the presence of crackles left lung base. The remainder exam was normal.

**Fig. 1:**
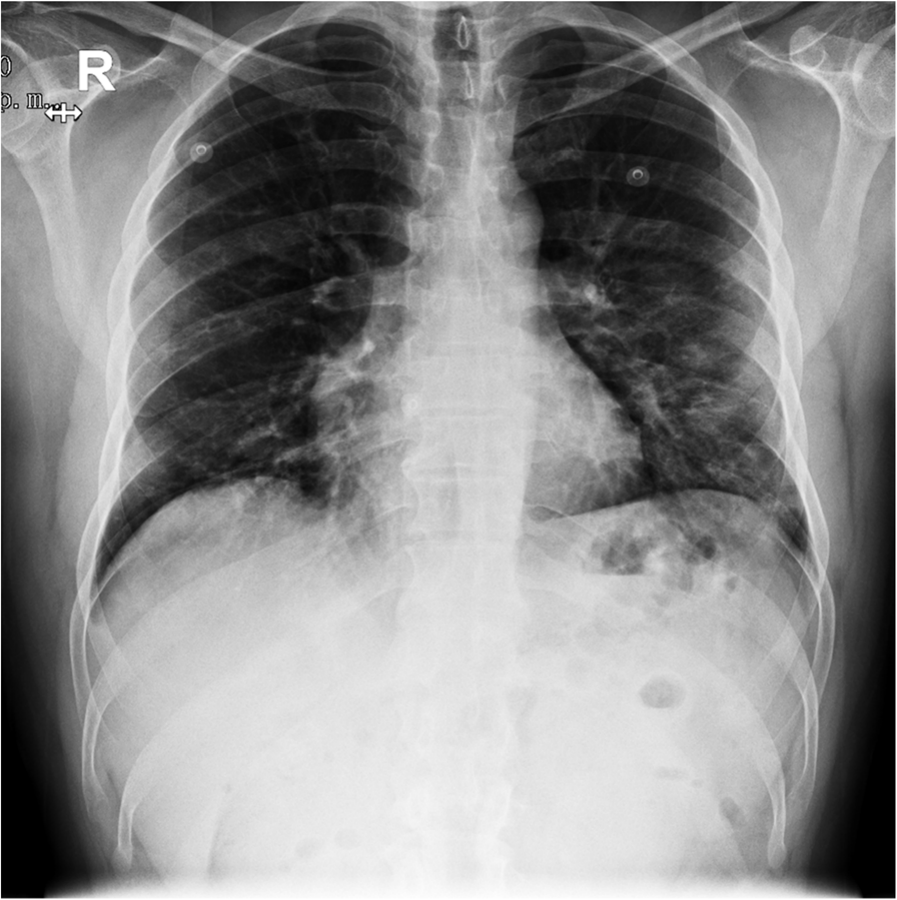
Chest X-ray showing multiple ground-glass bilateral infiltrates

**Fig. 2:**
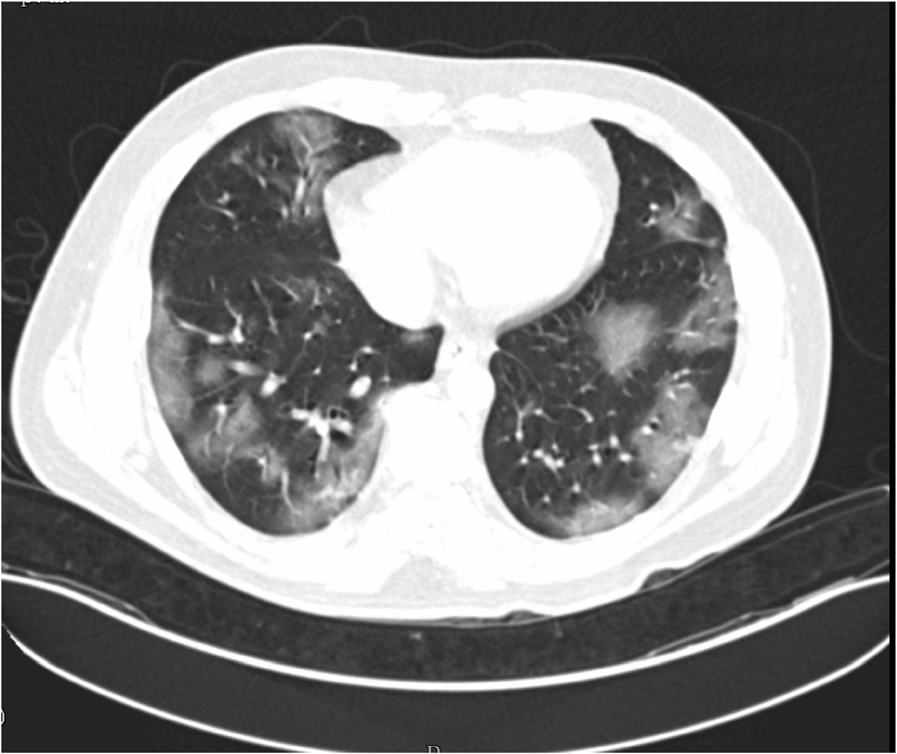
Chest CT scan showing multiple bilateral and peripheral ground-glass and consolidative pulmonary opacities

Initially, he was treated with IV metoclopramide, omeprazole 40 mg, ondansetron 8 mg, and oral frappe magaldrate/dimeticone (80/10 mg) without symptom improvement. Laboratory analysis revealed hyperglycemia (182 mg/dl), thrombocytopenia (81,000/mcl), leucopenia (4,000/mcl), and lymphopenia (700/mcl absolute count). The remaining results were normal (hemoglobin 16.2 g/l, hematocrit 48.3%, BUN 19.2, urea 41.1, serum creatinine 1.1 mg/dl, sodium 136.8 mEq/L, potassium 3.56 mEq/L). A chest X-ray (Fig. 1) revealed bilateral infiltrates. The thoracic CT scan revealed multiple zones of diffuse alveolar infiltrate across all segments of both lungs (Fig. 2).

SARS-CoV-2 PCR results were positive. The patient refused admission and was lost to follow-up.

### Case 2

A 43-year-old man was brought to the emergency department by his mother for altered mental status. She reported her son’s symptoms had started 3 days after his father was diagnosed with COVID-19. The patient presented with tachylalia, disorganized ideas, restlessness, delusions of grandeur, emotional lability, hetero-aggression, and aggression towards his mother. His mother revealed a past history of hetero-aggressive episodes which usually resolved during 48 h. Episodes did not affect social or professional functioning and were never medically evaluated.

Aside from his psychiatric symptoms, his vitals were normal, and his physical exam did not reveal any significant findings. Neurology and psychiatry were consulted, and due to known exposure, SARS-CoV-2 PCR was ordered.

While results were pending, we documented hypokalemia K 3.3, elevation of hepatic enzymes (aspartate aminotransferase 53 U/L, alanine aminotransferase 69 U/L), indirect hyperbilirubinemia (total bilirubin 2.07 mg/dl, indirect bilirubin 1.73 mg/dl, direct bilirubin 0.34 mg/dl), and elevated ferritin levels (595 ng/mL). The rest were within normal limits: C-reactive protein 1.8 mg/dL, erythrocyte sedimentation rate 10 mm/h, procalcitonin 0.05 ng/mL, and fibrinogen 297 g/L.

**Fig. 3:**
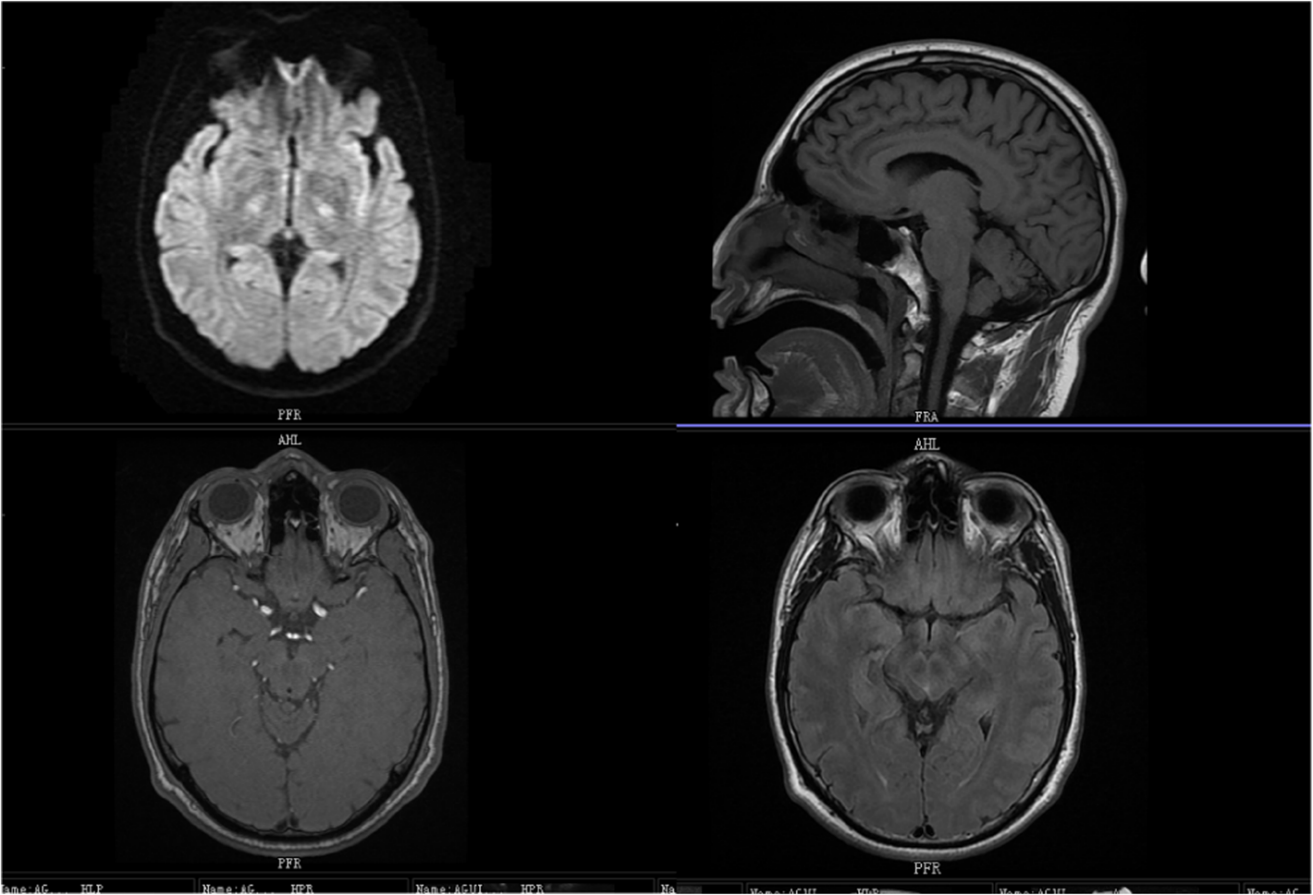
Brain MRI showing no signs of stroke or encephalitis

**Fig. 4:**
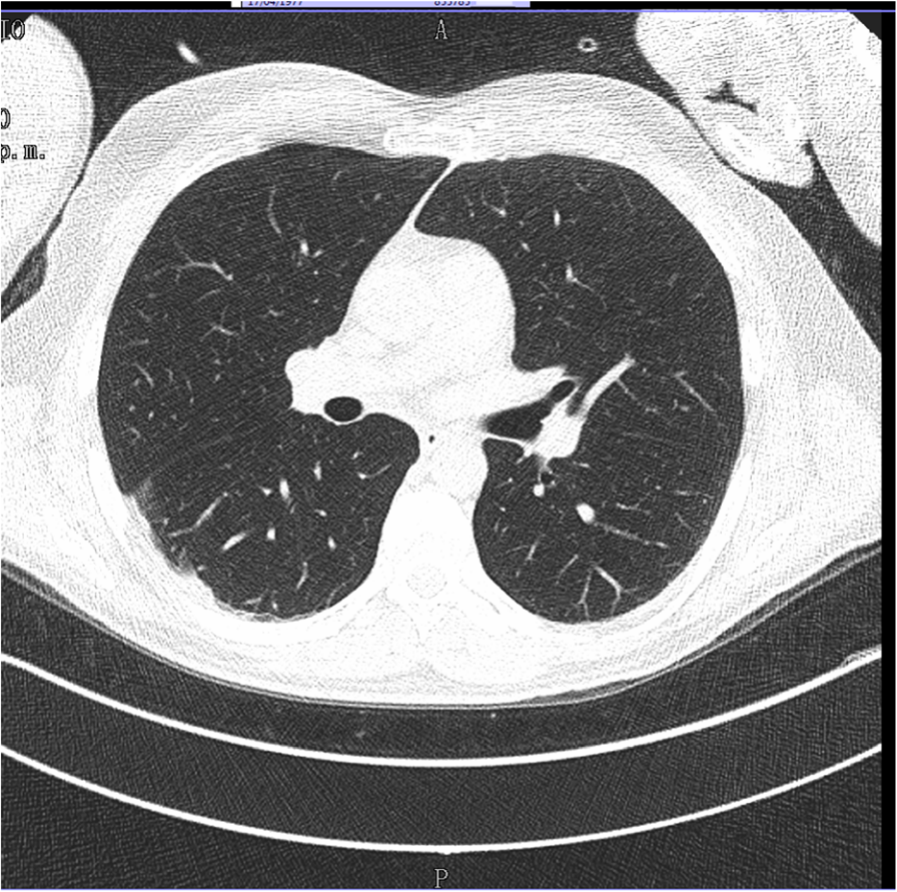
Chest CT scan not showing any infiltrates, consolidation images, or other signs of pneumonia; a nonsignificant single pleural nodule was identified

A contrast brain MRI (Fig. 3) and a thoracic CT (Fig. 4) were obtained to investigate for possible COVID-19-related changes such as encephalitis, stroke, or pulmonary changes. However, there were no significant findings.

A spinal tap and cytological testing yielded the following: transparent, with 0 leucocytes, 0 erythrocytes, and 0 crenocytes. Glucose 65.6 mg/dL, proteins 17.5 mg/dL, the Gram stain did not report any organisms, bacterial antigens were negative, and viral PCR did not detect any pathogen. Chinese ink was negative.

The PCR for SARS-CoV-2 was positive, and the patient was diagnosed with manic psychosis and COVID-19. Due to isolation precautions of the hospital, family members decided to take the patient home in a voluntary discharge. He will receive follow-up care by the neurologist and infectious disease specialist. The patient consented to the publishing of his experience.

## Discussion

### Case 1

Singultus or hiccups are caused by involuntary, myoclonic, and repetitive contractions of the diaphragm and intercostal muscles [9]. These coordinated contractions cause a rapid intake of air interrupted by closure of the glottis that results in the characteristic sound [10]. Singultus can be classified based on their duration. If the event lasts less than 48 h, it is called acute, persistent if last more than 48 h, and intractable if the attack lasts more than 30 days [9–11].

Although hiccups lasting > 48 h are rare, the workup should try to identify organic pathology. The main causes of prolonged hiccups can be divided in three groups: structural, infectious, and inflammatory disorders that impact either the central nervous system or the phrenic nerves or their branches [11, 12]. Searching for the cause of hiccups can be challenging due to the long course of nerves along the reflex pathway.

In this case, our patient had received pharmacological treatment for gastroesophageal causes of singultus with no improvement. The only remarkable finding during examination was crackles on lung auscultation. Similar to the case previously reported by Prince et al., where a 62-year-old woman presented with a 4-day history of hiccups, our patient had a chest X-ray and a CT scan showing ground-glass and consolidative pulmonary opacities compatible with SARS-CoV-2 pneumonia [13]. Thus, the most likely cause of the patient's symptoms was phrenic nerve inflammation secondary to COVID-19 pneumonia.

### Case 2

The second patient presented with no other symptom apart from an acute episode of psychosis, and we suspect this is a neuropsychiatric symptom of the disease. Several other cases have been reported; however, it is not clear how many of the others had lung involvement [7, 8, 14, 15]. Similar to the patient presented here, the three cases reported by Ferrando et al. did not show any respiratory tract involvement but did have elevated acute phase reactants and a positive SARS-CoV-2 PCR [14]. This differs from other patients experiencing psychotic symptoms because of fear for the disease but have been tested negative [15]. Even if Ferrando et al. suspect that the cytokine storm is involved in the pathophysiology behind these symptoms and this would explain this patient elevated ferritin and liver enzymes as well as the clear spinal tap, an EEG and the CSF SARS-CoV-2 PCR would be needed in order to rule out encephalitis [16] even if most patients with neurological symptoms with COVID-19 have been shown to have undetectable or very low levels of SARS-CoV-2 RNA in cerebrospinal fluid [17]. Another useful diagnostic test could have been oligoclonal band testing in serum and CSF, to identify whether the infection was primarily encephalitis or a result of systemic infection [18]. This could be useful to identify the presence of an escape syndrome variant, which is typically a phenomenon seen in patients with human immunodeficiency virus. The patients described by Ferrando et al. and this patient all presented with no other sign of organ involvement, and all four patients had no relevant past medical history. Escape syndrome described in HIV refers to patients with low plasmatic viral load but high CSF viral load and is the result of a well-controlled disease where virus migrate to the CSF and the patient develops new neuropsychiatric conditions [19]. Perhaps these cases represent viral escape when the patient’s immunological response is adequate. Further studies are needed in order to explain clearly the physiopathology of SARS-CoV-2. SARS-CoV-2 neuropsychiatric manifestations are uncommon, but should remain in an emergency physician’s differential especially if patients have been in close contact with the disease.

## Conclusions

We present two atypical cases of COVID-19. While most atypical manifestations have been described among children, older adults, and patients with multiple comorbidities, these cases include two young previously healthy men. To our knowledge, this is the second case of hiccups and COVID-19 reported in the literature and the fourth case of a psychotic episode as the only manifestation of the condition. While the patient presenting with hiccups has the expected physiopathology of SARS-CoV-2, it remains unclear if the psychosis presented in the second case is a result of encephalitis or cytokine storm. These two cases highlight the diversity of presentations of the condition and add to the growing knowledge bank about this virus.

## Data Availability

Please contact the author for data requests.
